# Functional Divergence in the Genus *Oenococcus* as Predicted by Genome Sequencing of the Newly-Described Species, *Oenococcus kitaharae*

**DOI:** 10.1371/journal.pone.0029626

**Published:** 2012-01-03

**Authors:** Anthony R. Borneman, Jane M. McCarthy, Paul J. Chambers, Eveline J. Bartowsky

**Affiliations:** The Australian Wine Research Institute, Glen Osmond, South Australia, Australia; King Abdullah University of Science and Technology, Saudi Arabia

## Abstract

*Oenococcus kitaharae* is only the second member of the genus *Oenococcus* to be identified and is the closest relative of the industrially important wine bacterium *Oenococcus oeni*. To provide insight into this new species, the genome of the type strain of *O. kitaharae*, DSM 17330, was sequenced. Comparison of the sequenced genomes of both species show that the genome of *O. kitaharae* DSM 17330 contains many genes with predicted functions in cellular defence (bacteriocins, antimicrobials, restriction-modification systems and a CRISPR locus) which are lacking in *O. oeni*. The two genomes also appear to differentially encode several metabolic pathways associated with amino acid biosynthesis and carbohydrate utilization and which have direct phenotypic consequences. This would indicate that the two species have evolved different survival techniques to suit their particular environmental niches. *O. oeni* has adapted to survive in the harsh, but predictable, environment of wine that provides very few competitive species. However *O. kitaharae* appears to have adapted to a growth environment in which biological competition provides a significant selective pressure by accumulating biological defence molecules, such as bacteriocins and restriction-modification systems, throughout its genome.

## Introduction

*Oenococcus kitaharae* is a lactic acid bacterium (LAB) that was recently isolated from composting distilled Shochu residue [1]. This species represents only the second member of the genus *Oenococcus* to be identified, with *Oenococcus oeni*, the founding member of this genus, being reclassified from *Leuconostoc oenos* by Dicks et al in 1995 [2]. Whereas little is known regarding the biology or ecology of *O. kitaharae*, *O. oeni* plays a pivotal role in the production of wine (its almost exclusive habitat) where it is responsible for performing malolactic fermentation (MLF) [3]. However, initial phenotypic comparisons would indicate that the environmental niche of *O. kitaharae* is very different to that of *O. oeni*. The two species display markedly different pH (6.0 to 6.8 versus 4.8, respectively) and temperature (30°C versus 22°C, respectively) optima and *O. kitaharae* is also incapable of growth in concentrations of ethanol routinely found in wine [1].

Given the importance of LAB to the food and beverage industries, it is not surprising that this group of bacteria has been the focus of extensive research, including several genome sequencing efforts. These have resulted in a broad phylogenetic genome sequencing survey of eight LAB genera (covering over 80 species) and, of particular relevance to the study of *O. kitaharae*, an intra-specific study of the genomes of three individual *O. oeni* isolates [4]–[6]. The results of these preliminary comparative genomic studies indicated that the LAB group harbours extensive genetic variation, such that even within single species such as *O. oeni*, coding potential can be over 10% different between any two strains [6].

In order to expand our understanding of the LAB group and to provide a point of comparison for understanding the genome dynamics of *O. oeni*, we have sequenced the genome of the *O. kitaharae* type strain DSM 17330 [1]. Comparisons between the *Oenococcus* genomes uncovered several genetic differences that have the potential to translate into important points of inter-specific phenotypic differentiation. These include several major metabolic differences such as the ability to ferment maltose, citrate and malate and the ability to synthesize specific amino acids such as L-arginine and L-histidine. In addition to these metabolic differences, the *O. kitaharae* genome also encodes many proteins involved in defence against both bacteriophage (restriction-modification and CRISPR) and other microorganisms (bacteriocins), and has had its genome populated by at least two conjugative transposons, which is in contrast to currently available genome sequences of *O. oeni* which lack the vast majority of these defence proteins. It therefore appears that the genome of *O. kitaharae* has been shaped by its need to survive in a competitive growth environment that is vastly different from that encountered by *O. oeni*, where environmental stresses provide the greatest challenge to growth and reproduction.

## Results

**Figure 1:**
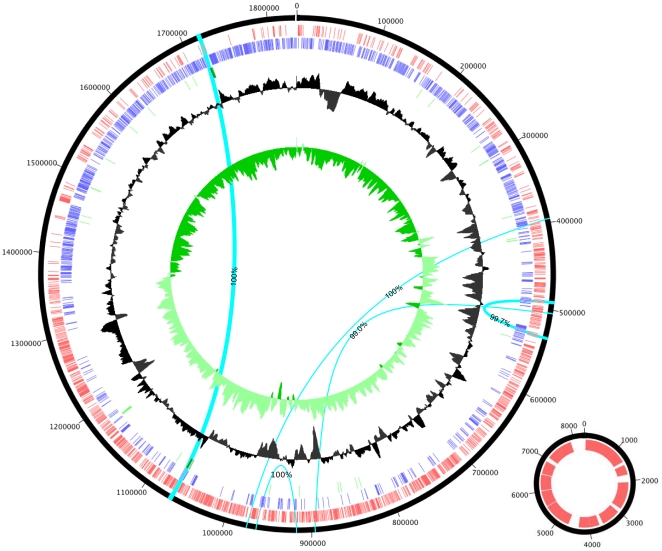
Circular representation of the chromosomal and plasmid replicons of *Oenococcus kitaharae*. Tracks represent (from largest to smallest) plus strand ORFs (red), minus strand ORFs (blue), RNA (tRNA light green, rRNA dark green), %GC and GC skew. The location of the five, two-copy repeats that are present in the *O. kitaharae* genome are also shown (light blue bars). Each of the five repeat groups are connected by arcs with the associated level of homology between each repeat listed.

The *O. kitaharae* DSM 17330 genome was assembled from 1×10^6^ Illumina paired-end reads (500 bp spacing) into 17 contigs, comprising 12 single-copy contigs in addition to five contigs that were present in two copies each (with three of these having 100% identity). Through the application of paired-end information and the precise order of the unique sequences bounding these repeats, these 17 contigs were able to be manually arranged into two replicons (Fig. 1). The first of these is a 1.84 Mb circular chromosome which, due to the presence of a highly repetitive repeat cluster that is associated with the coding region of a serine-repeat protein, contains one assembly gap. The second replicon is predicted to be an 8.3 kb plasmid, which, on the basis of sequencing coverage, is predicted to be present in low copy number (∼2 copies per cell, data not shown).

Genome annotation using a combination of Glimmer [7] and RAST [8] identified a total of 1833 predicted open reading frames (ORFs), four rRNA genes (two copies of each of the large and small ribosomal subunits), which are identical in sequence to the previously published ribosomal sequences for *O. kitaharae* [1], and 44 tRNA genes (see Table S1).

### Comparative genomics of *O. kitaharae* and LAB

**Figure 2:**
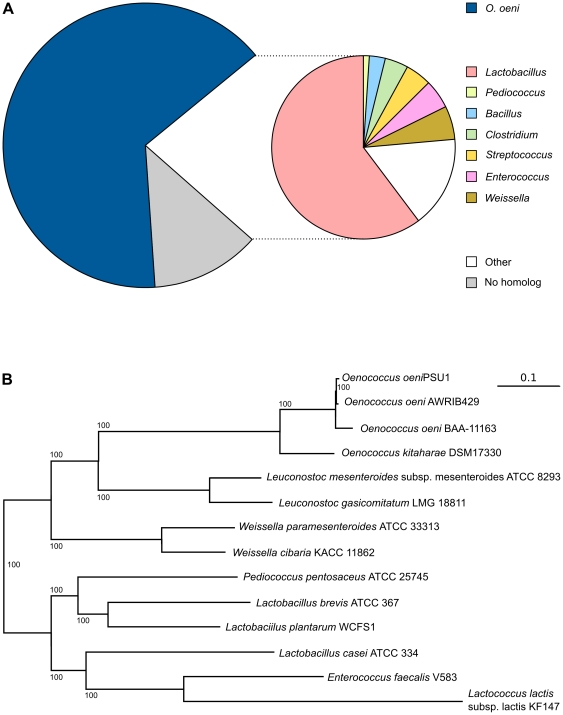
Evolutionary relationship of *Oenococcus kitaharae* and members of the LAB family. (**A**) The distribution of BLAST best-hits by genus for each ORF predicted in the *O. kitaharae* genome. (**B**) Whole genome phylogenetic relationship between *O. kitaharae* and other LAB based upon a conserved group of 95 proteins.

While the genome size of *O. kitaharae* is similar to that of *O. oeni* PSU1 (1.84 Mb vs 1.78 Mb), initial estimates of DNA-DNA relatedness were only 25% to 30% [1]. By direct comparison of the available genome sequences of *O. kitaharae* and *O. oeni*, a similar picture emerged. As expected, the majority of the ORFs predicted in the *O. kitaharae* genome (62%) have their closest homolog in *O. oeni* supporting the classification of *O. kitaharae* DSM 17330 within the *Oenococcus* genus (Fig. 2A). Of the remaining 703 *O. kitaharae* ORFs, 487 have at least one match in the Genbank non-redundant protein database, with LAB such as *Lactobacillus* spp, *Leuconostoc* spp, *Weissella* spp and *Lactococcus* spp predominating (Fig. 2A), while the remainder display no recognizable homolog and either represent novel protein sequences or false positives of the ORF prediction methodology applied.

In order to compare the phylogenetic position of *O. kitaharae* as determined from 16S rDNA sequencing [1] with that based upon whole genome data, homologs of each of the predicted ORFs of *O. kitaharae* were sought from whole genome sequence annotations of thirteen strains of LAB representing the genera *Enterococcus*, *Lactobacillus*, *Lactococcus*, *Leuconostoc*, *Oenococcus*, *Pediococcus* and *Weissella*. A total of 561 of the *O. kitaharae* ORFs were shown to be conserved across all thirteen strains (BLAST e-value<10^−20^, minimum 50% coverage of the query protein). Of these, 95 were then selected based on high sequence conservation and lack of potential paralogous sequences (see Table S2 for a complete list of the protein sequences used). Each of the subsequent individual protein alignments produced from these conserved groups of ORFs were then concatenated and used to construct a single maximum-likelihood phylogeny (Fig. 2B). The result of this analysis was consistent with phylogenies based upon 16S rDNA [1], [9], [10] and positions *O. kitaharae* as a clear sister species to *O. oeni* with both *Leuconostoc* spp. and *Weisella* spp. being the next closest evolutionary relatives.

#### Chromosomal elements of “foreign” origin

**Figure 3:**
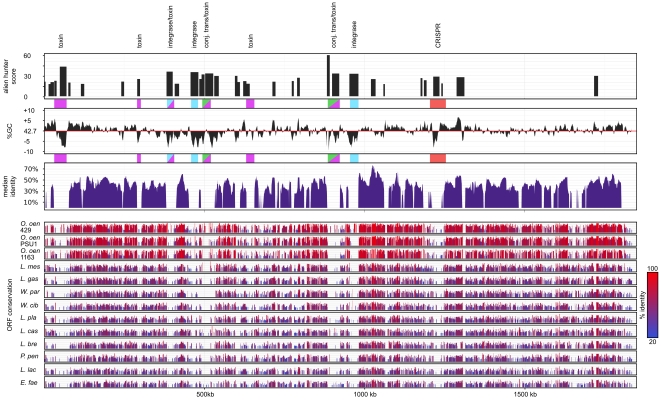
Conservation of the *Oenococcus kitaharae* genome. Homologs of each of the predicted *O. kitaharae* ORFs were sought from thirteen strains of LAB using BLAST and individual results are displayed for each strain color-coded by individual protein identity scores. In addition, an overall median identity was calculated by applying a sliding window of syntenic ORFs (n = 9, step = 1) to obtain a median percent identity for each strain with regions of low conservation highlighted (grey shading). Both the average GC percentage (5000 bp window, 200 bp step) and alien hunter foreign DNA likelihood scores [11] across the genome are also shown to compare areas of low sequence conservation with possible instances of HGT. The position of sequences associated with either toxin-antitoxin modules, phage integrase proteins, conjugative transposons or the CRISPR array are also shown.

The majority of the *O. kitaharae* genome is conserved with that of *O. oeni* with the exception of several large islands (Fig. 3). In all but a limited number of cases, these *O. kitaharae*-specific islands were also shown to lack identifiable homology with other species of LAB used in the phylogenetic constructions. In addition, the majority of these regions display a high probability of being acquired by horizontal gene transfer (HGT) [11] and would be expected to have entered the *O. kitaharae* genome via genetic elements such as bacteriophage or conjugative plasmids.

As previous investigations between strains of *O. oeni* have shown that non-conserved genomic islands can often be attributed to the differential presence of prophage elements [12], homology searches were used to identify classical prophage genes, such as those that encode conserved bacteriophage integrase and lyase proteins, in the genome of DSM 17330. While several *O. kitaharae* ORFs were found to be homologous to phage proteins (Table S1), each potential prophage region lacked the repertoire of proteins which would be expected for the presence of functional prophage elements [12]. In addition, in all but one case (the genomic region from 382388 bp–404717 bp), these genomic islands were not found downstream of tRNA genes as is observed for *O. oeni* phage, which use tRNA genes as attachment sites [12].

**Figure 4:**
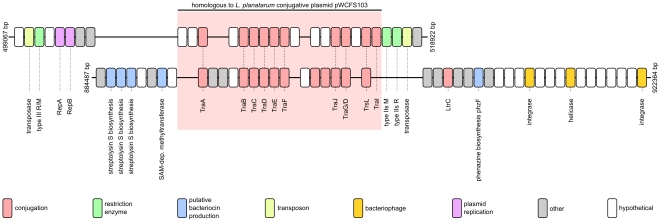
Schematic representation of two putative conjugative transposons present in the *Oenococcus kitaharae* genome. The ORFs present in each genomic element are colour coded by predicted function. The conserved conjugation-associated region present in the centre of each element is also highlighted (red shading).

Whereas the non-conserved genomic islands in the *O. kitaharae* genome do not appear to encode active phage, two of these islands have the hallmarks of genomically-integrated conjugative transposons (Fig. 4). Conjugative transposons are DNA elements that combine features of bacteriophage, transposons and conjugative plasmids [13]. These elements are often found integrated into the genome and are able to transpose to new sites in the host genome via the formation of a circular intermediate, which can sometimes replicate as a plasmid. As the name suggests, conjugative transposons also encode the ability to be horizontally transferred to new cells (and across species boundaries) via the classical conjugation pathway [13]. As yet, there have been no documented cases of conjugative transposons found in the genome of any strain of *O. oeni*.

The two potential conjugative transposons of *O. kitaharae* share a conserved core of 12 proteins. These proteins are predicted to encode the machinery necessary for conjugation and are highly homologous to a 12 kb region of the conjugative plasmid pWCFS103 from *Lactobacillus plantarum* [14]. In addition to the conserved conjugative core, one transposon is predicted to encode transposase-based integrative functions and the ability to replicate as a plasmid via the RepA and RepB plasmid replication proteins, whereas the other appears to use bacteriophage-based proteins for integration into the genome.

Accompanying the replicative functions of the *O. kitaharae* conjugative transposons, each element is predicted to encode several proteins that can be broadly categorised as functioning as part of “cellular defence” from either foreign DNA or from competition imposed by other microorganisms. One conjugative transposon is predicted to encode two separate restriction-modification (RM) systems (one combined Type III enzyme, and one Type IIs RM pair) and the other contains at least five ORFs that are potentially involved in the production of the antibacterial compounds (Fig. 4). In addition to the gene content of these potentially transmissible elements, the *O. kitaharae* DSM 17330 genome encodes another twelve proteins with putative roles in bacteriocin/antibacterial manufacture, transport or detoxification, four proteins involved in DNA RM and one CRISPR pathway array which, in other bacterial species, has been shown to provide a memory-based immunity to bacteriophage infection and possibly to the transmission of plasmid DNA (reviewed in [15]) (Table 1). All of these cellular defence mechanisms are lacking conserved homologs in the sequenced strains of *O. oeni*, with the exception of a single Type III RM enzyme which is found specifically in *O. oeni* strain AWRIB429 [6].

**Table 1:** Genes from *Oenococcus kitaharae* predicted to be involved in cellular defence.

Function	Description	ORF(s)
Bacteriocin production or immunity	Putative bacteriocin ABC transporter	OKIT_0291
	Bacteriocin immunity-associated integral membrane protein	OKIT_0292
	Bacteriocin, lactococcin 972 family	OKIT_0293
	Bacitracin transport ATP-binding protein	OKIT_0298
	Putative blasticidin S deaminase 2C	OKIT_0304
	Putative bacteriocin transport accessory protein	OKIT_0665
	Streptolysin S biosynthesis protein B (SagB)	OKIT_0885
	Streptolysin S biosynthesis protein C (SagC)	OKIT_0886
	Streptolysin S biosynthesis protein D (SagD)	OKIT_0887
	Phenazine biosynthesis protein PhzF	OKIT_0912
	Lactococcin A immunity protein	OKIT_0790
	Nisin transport protein	OKIT_0796
	Bacitracin export permease protein	OKIT_1723
	Putative bacteriocin ABC transporter	OKIT_1725
Restriction modification	Type III restriction enzyme2C res subunit:DEAD/DEAH box helicase2C N-terminal	OKIT_0515
	Type IIs modification methyltransferase	OKIT_0538
	Type IIs restriction endonuclease	OKIT_0539
	Type I restriction-modification system2C restriction subunit R (EC 3.1.21.3);Ontology_term = KEGG_ENZYME:3.1.21.3	OKIT_0971
	Type II restriction modification enzyme methyltransferase	OKIT_0974
	Type III restriction enzyme, restriction subunit	OKIT_0978
	5-methylcytosine-specific restriction enzyme^a^	OKIT_1348
CRISPR	CRISPR-associated protein2C SAG0894 family	OKIT_1269
	CRISPR-associated protein Cas1	OKIT_1270
	CRISPR-associated protein Cas2	OKIT_1271
	CRISPR-associated Csn2 family protein	OKIT_1272

a homolog found in *O. oeni* AWRIB429.

### Phenotypic differences attributable to genomic variation

In the initial characterization of *O. kitaharae* [1] several phenotypic traits were noted that differentiate this new species from *O. oeni*. Comparative genomics reveals a basis for some of these known differences while also suggesting several additional points of phenotypic differentiation.

#### Sugar utilization

One of the defining biochemical differences between *O. kitaharae* and *O. oeni* that was noted in its original isolation was the ability of *O. kitaharae* to produce acid from maltose [1]. This trait is rare in *O. oeni*, which is formally classified as maltose negative [16], [17]. By comparing available whole-genome annotations for *O. oeni* with *O. kitaharae* DSM 17330 [8], it was possible to identify several genes associated with sugar utilization that are differentially present across the species (Table 2). Of these, at least four genes which are present in *O. kitaharae*, but absent in the *O. oeni* genomes, are predicted to be involved in the utilization of maltose, providing a direct genetic basis for this phenotype. In addition to genes predicted to be involved in the species-specific utilization of maltose, there are several ORFs predicted to be involved in the metabolism of trehalose, D-gluconate, D-ribose and fructose that are specifically present in *O. kitaharae*. While the assimilation of these sugars is often carried out by specific strains of *O. oeni* [17], this genotypic data agrees well with biochemical tests performed previously that indicated that *O. kitaharae* was able to utilize all of these various carbon sources [1], [17].

**Table 2:** Carbohydrate utilization genes displaying inter-species differences.

Species	RAST Pathway	Description	ORF(s)
*O. kitaharae*	Fructooligosaccharide and Raffinose Utilization	MSM (multiple sugar metabolism) operon regulatory protein	OKIT_0495OKIT_0684
		Sucrose-6-phosphate hydrolase (EC 3.2.1.26)	OKIT_0688
*O. kitaharae*	Maltose & Maltodextrin Utilization	Maltose O-acetyltransferase (EC 2.3.1.79)	OKIT_0692
		Maltose/maltodextrin transport ATP-binding protein MalK (EC 3.6.3.19)	OKIT_0712
		Neopullulanase (EC 3.2.1.135)	OKIT_0711
		Pullulanase (EC 3.2.1.41)	OKIT_0709
*O. kitaharae*	D-ribose utilization	Ribose ABC transport system, ATP-binding protein RbsA (TC 3.A.1.2.1)	OKIT_0349
		Ribose ABC transport system, periplasmic ribose-binding protein RbsB (TC 3.A.1.2.1)	OKIT_0347
		Ribose ABC transport system, permease protein RbsC (TC 3.A.1.2.1)	OKIT_0348
*O. kitaharae*	Fructose utilization	PTS system, fructose-specific IIA component (EC 2.7.1.69)	OKIT_0249
		PTS system, fructose-specific IIB component (EC 2.7.1.69)	OKIT_0248
		PTS system, fructose-specific IIC component (EC 2.7.1.69)	OKIT_0250
*O. oeni*	COG3533	Arabinose-proton symporter	fig|203123.5.peg.226^a^
		L-arabinose isomerase (EC 5.3.1.4)	fig|203123.5.peg.224^b^
		L-ribulose-5-phosphate 4-epimerase (EC 5.1.3.4)	fig|203123.5.peg.223^b^
		Putative glycosyl hydrolase of unknown function (DUF1680)	YP_809865.1^c^
		Ribulokinase (EC 2.7.1.16)	YP_809879.1^c^
		Transcriptional repressor of arabinoside utilization operon, GntR family	YP_809878.1^c^
		Xyloside transporter XynT	YP_810752.1^c^; fig|203123.5.peg.206^a^

a RAST protein ID (not annotated in *O. oeni* PSU-1 Genbank submission).

b RAST protein ID (pseudogene in *O. oeni* PSU-1, full ORF present in other strains of *O. oeni*).

c *O. oeni* Genbank protein ID from genome accession number NC_008528.1.

In addition to those genes that are specifically present in *O. kitaharae* DSM 17330, several were identified that were present only in strains of *O. oeni* and which are predicted to be involved in the uptake and metabolism of arabinose and xylose (Table 2). This is consistent with the inability of *O. kitaharae* to produce acid from either L-arabinose or D-xylose, two biochemical reactions that many strains of *O. oeni*, including those for which genome sequence are available often perform [1], [17].

#### Amino acid biosynthesis

Both *O. oeni* and *O. kitaharae* are fastidious microorganisms that require many exogenous vitamins and amino acids. However, it appears that the *O. kitaharae* genome encodes biosynthetic pathways for at least two amino acids, arginine and histidine, which are lacking in *O. oeni* [18].

The *O. kitaharae* DSM 17330 genome encodes the six genes necessary for the production of arginine from glutamate via the ornithine/carbamoyl-phosphate (CP) pathway, in addition to encoding a second set of carbamoyl-phosphate synthase (CPSase) proteins (Table 3). As CP is an important intermediate in both the arginine and pyrimidine biosynthetic pathways, many bacteria, such as *Lactobacillus plantarum*, contain two completely separate sets of CPSase proteins [19]. In this situation, one protein is encoded in an operon with genes involved in arginine biosynthesis and regulated by arginine, while a second gene is located in the pyrimidine biosynthetic operon and regulated by exogenous pyrimidines. *O. kitaharae* contains both sets of CPSase enzymes while the *O. oeni* genomic sequences are predicted to encode only the single pyrimidine-associated CPSase [19].

**Table 3:** Genes involved in arginine and histidine biosynthesis in *O. kitaharae*.

RAST Pathway	Description	ORF(s)
Arginine biosynthesis	Acetylglutamate kinase (EC 2.7.2.8)	OKIT_0634
	Acetylornithine aminotransferase (EC 2.6.1.11)	OKIT_0630
	Glutamate N-acetyltransferase (EC 2.3.1.35)	OKIT_0629
	N-acetyl-gamma-glutamyl-phosphate reductase (EC 1.2.1.38)	OKIT_0628
	N-acetylglutamate synthase (EC 2.3.1.1)	OKIT_0629
	Ornithine carbamoyltransferase (EC 2.1.3.3)	OKIT_0631
	Carbamoyl-phosphate synthase small chain (EC 6.3.5.5)	OKIT_0632
	Carbamoyl-phosphate synthase large chain (EC 6.3.5.5)	OKIT_0633
Histidine biosynthesis	phosphoribosyl-AMP cyclohydrolase( EC:3.5.4.19 )	OKIT_1691
	Phosphoribosyl-ATP pyrophosphatase (EC 3.6.1.31)	OKIT_1690
	Imidazoleglycerol-phosphate dehydratase (EC 4.2.1.19)	OKIT_1695
	Imidazole glycerol phosphate synthase cyclase subunit (EC 4.1.3.-)	OKIT_1692
	Imidazole glycerol phosphate synthase amidotransferase subunit (EC 2.4.2.-)	OKIT_1694
	Histidinol-phosphate aminotransferase (EC 2.6.1.9)	OKIT_1689
	Histidinol-phosphatase (EC 3.1.3.15)	OKIT_1699
	Histidinol dehydrogenase (EC 1.1.1.23)	OKIT_1696
	ATP phosphoribosyltransferase regulatory subunit (EC 2.4.2.17)	OKIT_1698
	ATP phosphoribosyltransferase catalytic subunit (EC 2.4.2.17)	OKIT_1697
	Phosphoribosylformimino-5-aminoimidazole carboxamide ribotide isomerase (EC 5.3.1.16)	OKIT_1693

*O. kitaharae* also appears to encode all of the enzymes necessary for the synthesis of histidine from the pentose phosphate pathway intermediate 5-phosphoribosyl 1-pyrophosphate (PRPP) (Table 3). These 11 genes lie adjacent to each other in the *O. kitaharae* DSM 17330 genome while *O. oeni* genome sequences lack this entire complement of enzymes.

#### Organic acid metabolism

Many strains of *O. oeni* are capable of fermenting citrate when additional sources of fermentable carbon sources are also present [20], [21]. Several genes in the *O. oeni* PSU-1 genome have been identified previously that would provide this strain with the ability to convert citrate to pyruvate [4] (Table 4). These genes are absent from the *O. kitaharae* DSM 17330 genome leading to the prediction that, unlike *O. oeni*, this strain would lack the ability to ferment this organic acid.

**Table 4:** Genes involved in citrate utilization in *O. oeni*.

RAST Pathway	Description	ORF(s)
Citrate Metabolism, Transport, and Regulation	2-(5″-triphosphoribosyl)-3′-dephosphocoenzyme-A synthase (EC 2.7.8.25)	YP_810049.1^a^
	Apo-citrate lyase phosphoribosyl-dephospho-CoA transferase (EC 2.7.7.61)	YP_810048.1^a^
	Citrate lyase alpha chain (EC 4.1.3.6)	YP_810047.1^a^
	Citrate lyase beta chain (EC 4.1.3.6)	YP_810046.1^a^
	Citrate lyase gamma chain, acyl carrier protein (EC 4.1.3.6)	YP_810045.1^a^
	Citrate lyase transcriptional regulator CitI	YP_810041.1^a^
	Oxaloacetate decarboxylase involved in citrate fermentation (EC 4.1.1.3)	YP_810042.1^a^
	[Citrate [pro-3S]-lyase] ligase (EC 6.2.1.22)	YP_810044.1^a^

a *O. oeni* Genbank protein ID from genome accession number NC_008528.1.

**Figure 5:**
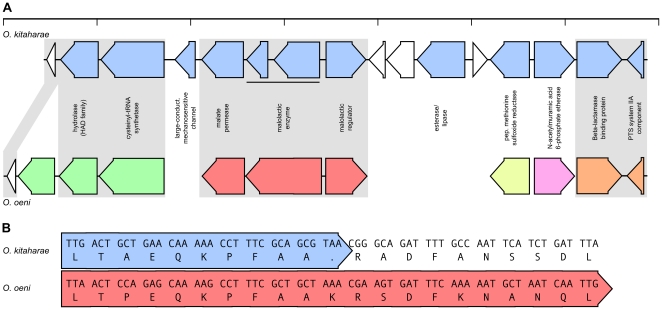
The malate operon of *Oenococcus kitaharae*. (**A**) Schematic representation of the genomic region surrounding the non-functional malate operon in *O. kitaharae*. *O. kitaharae* ORFs (blue) are shown above their orthologs from *O. oeni* with regions of microsynteny indicated by the differential shading of the *O. oeni* ORFs (green, red, yellow, pink and orange). (**B**) Partial alignment of the ORF which encodes malate enzyme *O.* oeni (red) with the homologous region from *O. kitaharae* (blue). Both the DNA and predicted protein sequences are listed.

One of the key defining biochemical features that separates *O. oeni* from *O. kitaharae* is the ability to perform malolactic fermentation. Malolactic fermentation has been shown to require the action of three proteins, a malate permease, which transports malate into the cell, the malolactic enzyme, which is responsible for converting malic acid into lactic acid, and a regulatory protein for these two downstream genes [22]. Surprisingly, the *O. kitaharae* genome was shown to contain genes that are orthologous to those which encode all three of these activities in *O. oeni* (Fig. 5A). It was subsequently shown that, while the sequences of all three genes are present in the *O. kitaharae* genome, the gene encoding malolactic enzyme contained a nonsense mutation that would prematurely truncate the protein coding region (Fig. 5B). The alteration of a single base in this premature stop codon would be sufficient to restore the full-length malolactic enzyme coding region (Fig. 5B) that is highly conserved with malolactic enzymes from many bacteria (Fig. S1). Furthermore, the *O. kitaharae* gene was shown to have a low ratio of non-synonymous to synonymous mutations (*dN*/*dS* = 0.0123) when compared with its *O. oeni* homologue. This would indicate that there has been limited opportunity for the unconstrained accumulation of synonymous mutations in the two fragments of the malolactic enzyme coding region in *O. kitaharae* (as would be expected in a non-functional gene undergoing random drift). It is therefore likely that the conversion of the malolactic enzyme to a pseudogene is a very recent event in *O.* kitaharae and it may be possible to obtain a functional enzyme through reversion of the nonsense mutation or to find a functional malate pathway in strains of *O. kitaharae* other than DSM 17330.

## Discussion

*O. kitaharae* and *O. oeni* comprise the only known members of their genus. Sequencing of the *O. kitaharae* DSM 17330 genome has provided important insights into the genetic diversity across this genus. These two species of *Oenococcus* appear to inhabit significantly different ecological niches, with *O. oeni* being found almost exclusively in the highly stressful environment of wine whereas *O. kitaharae* was isolated from a composting shochu residue of unknown nutrient composition. Accordingly, the two species have accumulated genetic adaptations that reflect different metabolic needs and environmental constraints.

Although little is known regarding the exact nutrient profile of the residue from which *O. kitaharae* was isolated, the average composition of the major wine metabolites are well known. Finished wine has little or no glucose, fructose or maltose but does contain significant quantities of arabinose and xylose [23], [24]. Many strains of *O. oeni* are capable of exploiting these carbohydrates, and contain genes whose biochemical functions are consistent with this ability, but, in all but a limited number of cases [16], [17], cannot utilize maltose. In comparison, *O. kitaharae* DSM 17330 lacks the genes required for the use of arabinose and xylose but has the ability to utilize the maltose that would be present in the feedstocks, such as barley, which are used in the production of shochu.

In addition, while little is known regarding the amino acid profile of the shochu residue in which *O. kitaharae* was isolated, it would be predicted that these feedstocks would generally be lower in arginine and histidine than wine (where they are often amongst the most prevalent amino acids [24]) given the presence of the biosynthetic pathways for both of these amino acids in *O. kitaharae*. Interestingly, both the histidine and arginine biosynthetic pathways display a scattered pattern of presence throughout the LAB phylogeny with only a limited number of species within a genus possessing these pathways (Fig. S2). It appears that there must be significant selective pressure working for and against these biosynthetic pathways in an environmentally-dependent manner across the LAB. For *O. kitaharae*, the evolutionary origins of both pathways are more consistent with loss of these enzymes followed by horizontal gain from a *Lactobacillus*-related species (Fig. S2).

Wine represents a harsh growth environment in which only a select few species of bacteria are capable of growth to significant levels [25]. *O. oeni* is therefore faced with little competition from other species of bacteria during its growth and its genome is almost devoid of proteins that are involved in defence against other bacteria or even to invasion by bacteriophage. In contrast, the *O. kitaharae* genome contains numerous proteins that potentially provide a selective advantage over other bacteria (bacteriocins/antimicrobials), to defend against attack by other species of bacteria (bacteriocin immunity proteins) and to also defend against invasion by foreign DNA, such as that introduced by bacteriophage (restriction-modification systems and the CRISPR element). Although the biological diversity of composting residue in which *O. kitaharae* was not formally evaluated, it can be assumed that there was sufficient microbiological competition for resources to justify the selective advantage for the presence of these defence compounds. This argument is further supported by the fact that at least two other novel species of LAB have been isolated from this environment in addition to many other species of LAB that have been shown to be present during the shochu production process [1], [26]–[29]. *O. kitaharae* has therefore evolved to compete in a mixed-species environment whereas *O. oeni* has adapted to a niche in which the extreme nature of the growth substrate has removed the majority of biological competition.

Whereas the applicability of *O. kitaharae* for use as an industrial species is yet to be determined, it could prove useful for the development of improved strains of its relative *O. oeni*. Despite the environment of wine providing protection from competition for *O. oeni*, it is still argued that many instances of failed malolactic fermentations are due to the action of bacteriophage on susceptible strains [30], [31]. If it were possible to move genes, such as those of the CRISPR array, from *O. kitaharae* into *O. oeni* via the conjugation machinery which is predicted to be present in *O. kitaharae*, this could provide a non-GM means of equipping industrial *O. oeni* strains with general resistance to bacteriophage infection. Likewise, if the genes involved in antimicrobial production can be transferred to *O. oeni*, these strains could limit potential negative impacts on wine quality due to the growth of spoilage bacteria such as *Pediococcus* spp, *Lactobacillus* spp and acetic acid bacteria, and may provide the means to reduce the amount of sulfite that is currently used for the microbial stabilization of wine [32]. The use of genes from *O. kitaharae* may therefore allow the production of strains of *O. oeni* which are not only able to thrive in the harshness of the wine environment, but are more resistant to potential biological competition from bacteriophage or other microorganisms.

## Materials and Methods

### DNA isolation and sequencing

*O. kitaharae* DSM 17330 was obtained from DSMZ (Germany) and was grown in modified MRS media (Amyl, Australia). Genomic DNA was isolated using standard phenol-chloroform extractions. Sequencing was performed on an Illumina GAIIx using 2×100 bp paired-end ends with an average library size of 500 bp (Ramacioitti Centre, NSW, Australia).

### Genome assembly

A total of 990,000 reads (∼50-fold expected genome coverage) were randomly selected and assembled using MIRA (version 3.2.1). The MIRA output was imported into Seqman Pro (DNAstar, Madison, WI) for manual alignment and editing of the assembly. This Whole Genome Shotgun project has been deposited at DDBJ/EMBL/GenBank under the accession AFVZ00000000. The version described in this paper is the first version, AFVZ01000000.

### Genome Annotation

Gene predictions were made using Glimmer 3.02 [7]. Gene functional annotations were performed using the RAST server [8] and BLAST [33] with comparisons to the non-redundant Genbank database. Predictions of genomic regions likely to have been acquired by horizontal gene transfer were calculated using Alien Hunter [11]. dN/dS ratios were calculated using Pal2nal [34]. Circular genome plots were compiled using Artemis [35] and DNAplotter [36].

Comparisons to the various LAB genomes were performed using BLAST [33] and custom written scripts. For phylogenetic analysis, proteins used for the analysis were first screened to ensure that they were conserved (minimum 60% identity when compared to the homologous *O. kitaharae* protein) in all of the LAB genomes used in this study (See Table S2). Next, proteins which had potential paralogs (which could confound the phylogeny) were identified by assigning each protein to specific orthoMCL [37] clusters and then only retaining those groups of orthologs in which each protein was the only member of a particular orthoMCL group. Individual protein alignments were then performed on each set of homologous sequences using Muscle [38]. These individual alignments were then concatenated into a single large sequence for each strain which was used to construct a maximum-likelihood phylogenetic tree using PhyML [39].

## Supporting Information

Figure S1 **Amino acid alignment of malolactic enzymes from various species of lactic acid bacteria.** Amino acid sequences were aligned using ClustalX and conserved residues (>60%) are highlighted (black shading). The position of the in-frame stop-codon in *O. kitaharae* is also highlighted (red shading).
(TIF)

Figure S2 **Phylogenetic relationship of the histidine and arginine operons of** ***O. kitaharae*** **.** ORFs from each pathway in each species (if present) were concatenated prior to alignment. Each maximum-likelihood tree is presented for comparison at the same scale as the full tree which is comprised of 95 conserved ORFS from 13 species of lactic acid bacteria.
(TIF)

Table S1 ***Oenococcus kitaharae*** **genome annotation.** *O. kitaharae* ORFs as predicted by Glimmer [7] are matched against an automated annotation and functional prediction performed using RAST [8]. Comparative analysis of each ORF was also performed using BLASTp comparisons against *O. oeni* PSU1 [33] with the comparisons parameters listed for each *O. kitaharae* ORF and its closest *O. oeni* PSU1 match (if present).
(XLS)

Table S2 **Evolutionarily conserved ORFs in lactic acid bacteria (LAB).** Homologs of each of the predicted *O. kitaharae* ORFs were sought from thirteen strains of LAB using BLASTp [33]. Evolutionarily conserved proteins were classified as exhibiting sequence conservation across all fourteen species (minimum 60% identity when compared to the homologous *O. kitaharae* protein). This list was further refined by mapping each ORF from each species to an orthoMCL group [37] and only retaining those ORFs which were found be be unique within their particular orthoMCL cluster (removes the potential for paralogous ORFs to interfere with the phylogeny).
(XLS)
