# Supplementary figures and images for: Functional Divergence in the Genus Oenococcus as Predicted by Genome Sequencing of the Newly-Described Species, Oenococcus kitaharae
Source: PLoS One. 2012 Jan 3;7(1):e29626. doi: 10.1371/journal.pone.0029626 (PMC3250461; doi:10.1371/journal.pone.0029626)

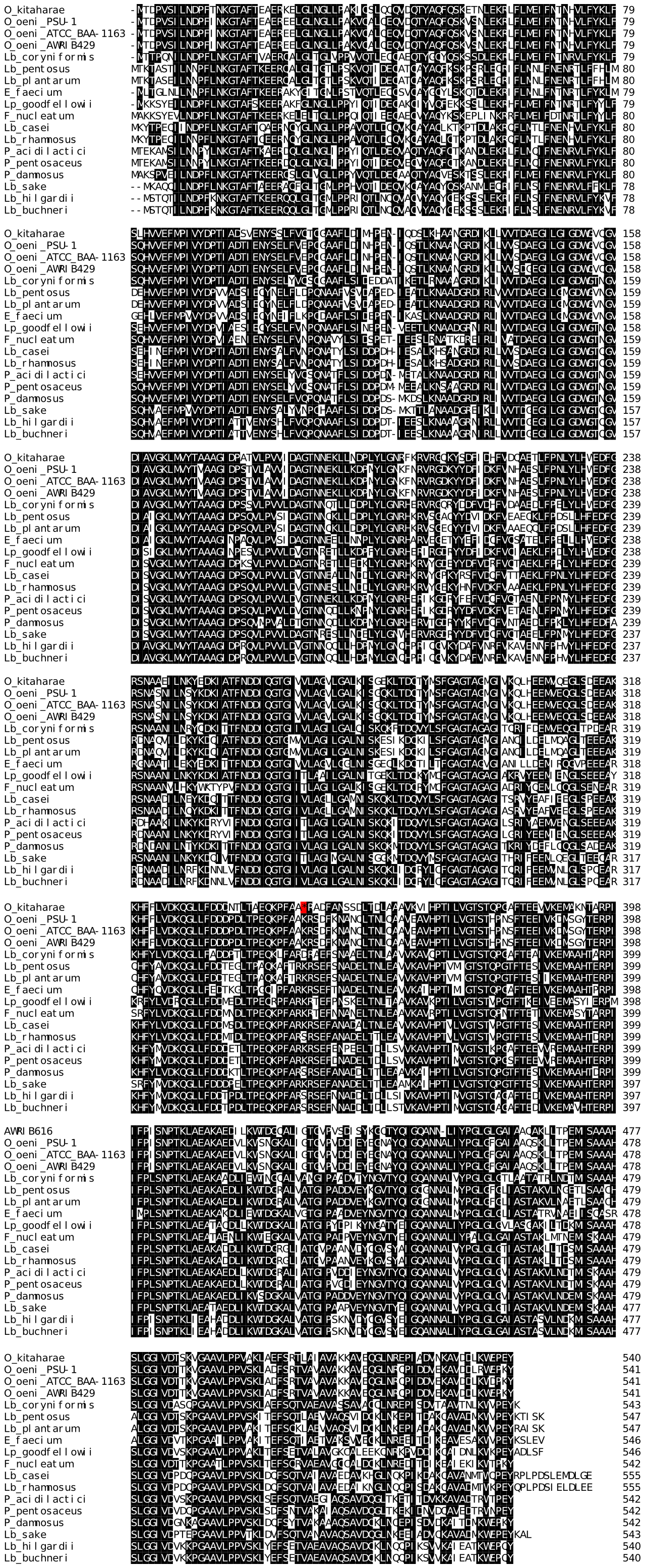

Supplement: Figure S1 — Amino acid alignment of malolactic enzymes from various species of lactic acid bacteria. Amino acid sequences were aligned using ClustalX and conserved residues (>60%) are highlighted (black shading). The position of the in-frame stop-codon in O. kitaharae is also highlighted (red shading). (TIF) [file pone.0029626.s001.tif]

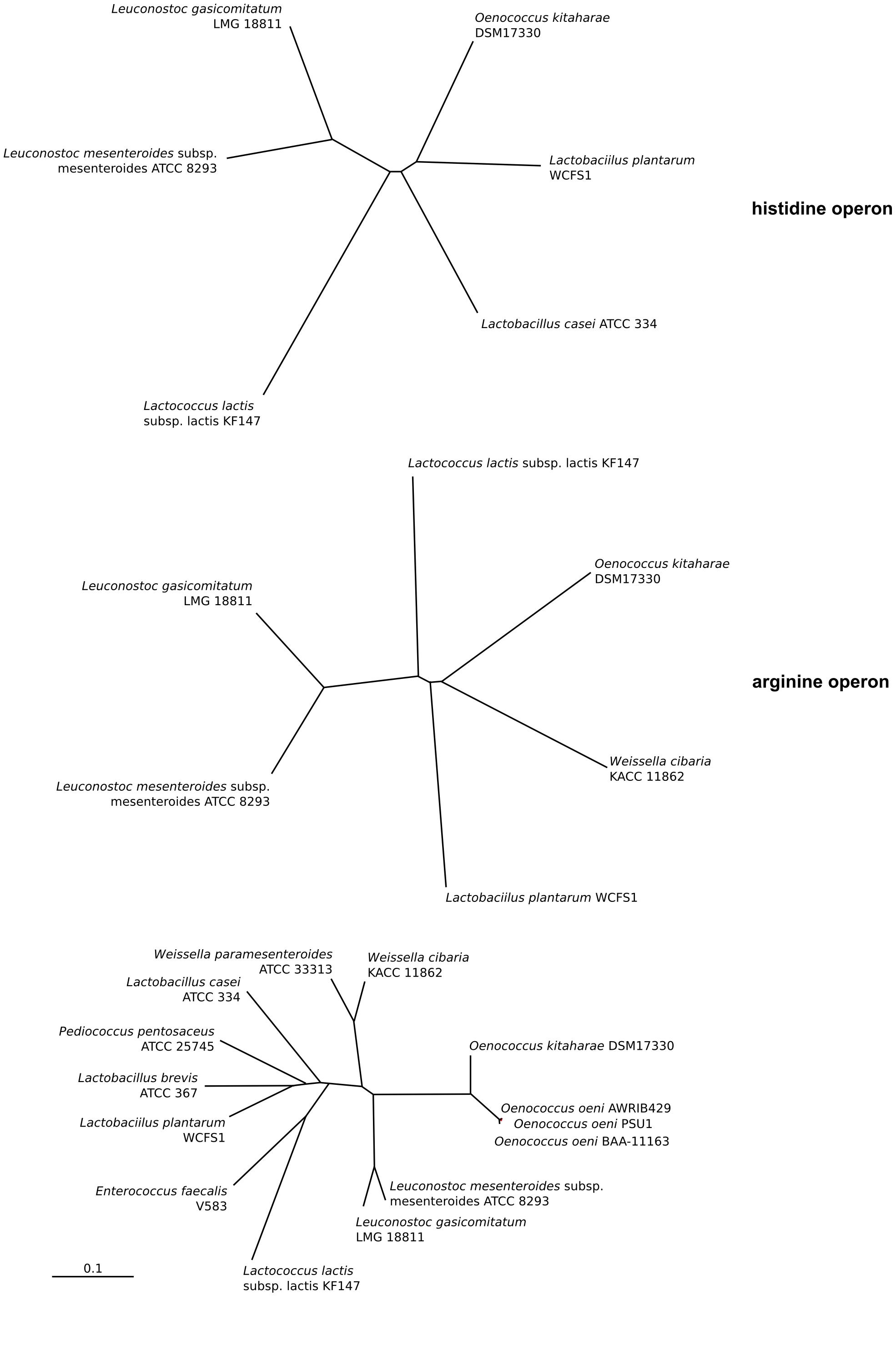

Supplement: Figure S2 — Phylogenetic relationship of the histidine and arginine operons of O. kitaharae . ORFs from each pathway in each species (if present) were concatenated prior to alignment. Each maximum-likelihood tree is presented for comparison at the same scale as the full tree which is comprised of 95 conserved ORFS from 13 species of lactic acid bacteria. (TIF) [file pone.0029626.s002.tif]
